# Complete Genome Sequence of *Adlercreutzia* sp. Strain 8CFCBH1, a Potent Producer of Equol, Isolated from Healthy Japanese Feces

**DOI:** 10.1128/MRA.01240-20

**Published:** 2020-12-03

**Authors:** Yusuke Ogata, Mitsuo Sakamoto, Moriya Ohkuma, Masahira Hattori, Wataru Suda

**Affiliations:** a Laboratory for Microbiome Sciences, RIKEN Center for Integrative Medical Sciences, Yokohama, Japan; b Microbe Division/Japan Collection of Microorganisms, RIKEN BioResource Research Center, Tsukuba, Japan; c PRIME, Japan Agency for Medical Research and Development, Tsukuba, Japan; d Graduate School of Advanced Science and Engineering, Waseda University, Tokyo, Japan; University of Rochester School of Medicine and Dentistry

## Abstract

Here, we report the complete genome sequence of *Adlercreutzia* sp. strain 8CFCBH1, which was isolated from a Japanese fecal sample. The genome analysis revealed that the 8CFCBH1 strain potentially produces (*S*)-equol.

## ANNOUNCEMENT

Certain gut bacteria have the ability to metabolically convert daidzein in soybeans and related foods to equol, which has been reported to have beneficial effects on some symptoms (1, 2). There have been reports on genome sequences of several equol-producing bacteria (3–6), and we report the genome of an additional *Adlercreutzia* sp. strain, 8CFCBH1, isolated from a 55-year-old healthy Japanese female.

All of the experiments were approved by the RIKEN ethics committee (approval Tsukuba 27-1), and manufacturer’s instructions and default parameters were used for all experiments and software, respectively, unless otherwise specified. A fecal sample (0.5 g) was suspended in 4.5 ml of prereduced phosphate-buffered saline (PBS). The diluted fecal sample was plated onto Columbia blood agar supplemented with 5% (vol/vol) horse blood for 2 to 4 days of incubation at 37°C under a H_2_/CO_2_/N_2_ (1:1:8, by volume) gas mixture. The partial 16S rRNA sequencing of grown colonies was performed using the BigDye Terminator v. 3.1 cycle sequencing kit with an Applied Biosystems SeqStudio genetic analyzer (Thermo Fisher Scientific). After the sequencing, we performed a similarity search using NCBI nucleotide BLAST with the rRNA/internal transcribed spacer (ITS) database, and we identified the isolates as an *Adlercreutzia* sp. The 8CFCBH1 strain was then grown in 500 ml of Gifu anaerobic medium (GAM broth; Nissui) for 7 days at 37°C to prepare the genomic DNA. The DNA extraction was performed based on enzymatic lysis as described previously (7). The DNA sequencing was performed using both Illumina MiSeq and PacBio Sequel platforms. The library for Illumina MiSeq 2 × 300-bp paired-end sequencing was prepared using the TruSeq DNA PCR-free kit (target length, 550 bp), and all of the MiSeq reads were trimmed and filtered with a >20 quality value using FASTX-toolkit (v. 0.0.13) (http://hannonlab.cshl.edu/fastx_toolkit). The library for PacBio Sequel sequencing was prepared using the SMRTbell v. 2.0 template preparation kit (target length, 10 to 15 kbp) without DNA shearing. After removal of the internal control and adaptor trimming by Sequel, error correction of the trimmed reads was performed using Canu (v. 1.8) (8) with additional options (corOutCoverage=10000, corMinCoverage=0, corMhapSensitivity=high), *de novo* hybrid assembly of the filter-passed MiSeq reads and the corrected Sequel reads was performed using Unicycler (v. 0.4.8) (9), including a check of overlapping and circularization, and a circular contig was generated. The gene prediction and genome annotation of the generated contig were performed using DFAST (v. 1.2.4) (https://dfast.nig.ac.jp). We obtained a total of 872,477,675 bases from 1,467,011 filter-passed MiSeq paired-end reads with an average length of 299.3 bp and a total of 209,990,690 bases from 16,508 corrected Sequel reads with an *N*_50_ value of 19,523 bp.

The *Adlercreutzia* sp. strain 8CFCBH1 chromosome was 2,908,404 bp long with a GC content of 63.0% and contained 2,452 protein-coding genes, 50 tRNA genes, and 3 5S rRNA, 3 16S rRNA, and 3 23S rRNA genes. The 16S rRNA sequence had the greatest similarity to that of Adlercreutzia equolifaciens subsp. *equolifaciens* JCM 14793^T^ (DSM 19450^T^) with 98.7% identity in the NCBI rRNA/ITS database (10 January 2020); however, the average nucleotide identity (ANI) was 93.3% by the ANI calculator (https://www.ezbiocloud.net/tools/ani) and the 40-housekeeping-gene similarity was 95.3% by nucmer (v. 4.0.0 beta2) (10) with respect to the JCM 14793^T^ chromosome. In addition, an alignment of the two chromosomes by Mauve (v. 2.0) (http://darlinglab.org/mauve/mauve.html) revealed a large inversion of ∼1.7 Mb in addition to >400 indels of >1 kb between the chromosomes, and the 8CFCBH1 strain lacked a gene for dihydrodaidzein racemase, one of four genes involved in equol production contained in the JCM 14793^T^ chromosome (11).

### Data availability.

The complete genome sequence of *Adlercreutzia* sp. strain 8CFCBH1 was deposited in DDBJ/ENA/GenBank under the accession no. AP022829, which is linked to the BioProject accession no. PRJDB9316, the BioSample accession no. SAMD00204449, and the DDBJ Sequence Read Archive accession no. DRR207927 and DRR207928.
